# Supplementary material for: Bacteroides muris sp. nov. isolated from the cecum of wild-derived house mice
Source: Arch Microbiol. 2022 Aug 8;204(9):546. doi: 10.1007/s00203-022-03148-6 (PMC9360105; doi:10.1007/s00203-022-03148-6)
Supplement: Supplementary file 1 — Supplementary file1 (PDF 1582 KB) [file 203_2022_3148_MOESM1_ESM.pdf]

Bacteroides muris sp. nov. isolated from the cecum of wild-derived house mice

Archives of Microbiology

Supplementary Information

Hanna Fokt[1], Rahul Unni[1,2], Urska Repnik[3], Ruth A Schmitz[4], Marc Bramkamp[3,4], John F. Baines[1,2], Daniel Unterweger[1,2]

[1]Max Planck Institute for Evolutionary Biology, 24306 Plön, Germany

[2]Institute for Experimental Medicine, Kiel University, 24105 Kiel, Germany

[3]Central Microscopy Facility, Kiel University, 24118 Kiel, Germany

[4]Institute for General Microbiology, Kiel University, 24118 Kiel, Germany

Corresponding authors

Daniel Unterweger

unterweger@evolbio.mpg.de

John F. Baines

baines@evolbio.mpg.de

Figure S1, S2, S3, S4, S5

Table S1, S2, S3

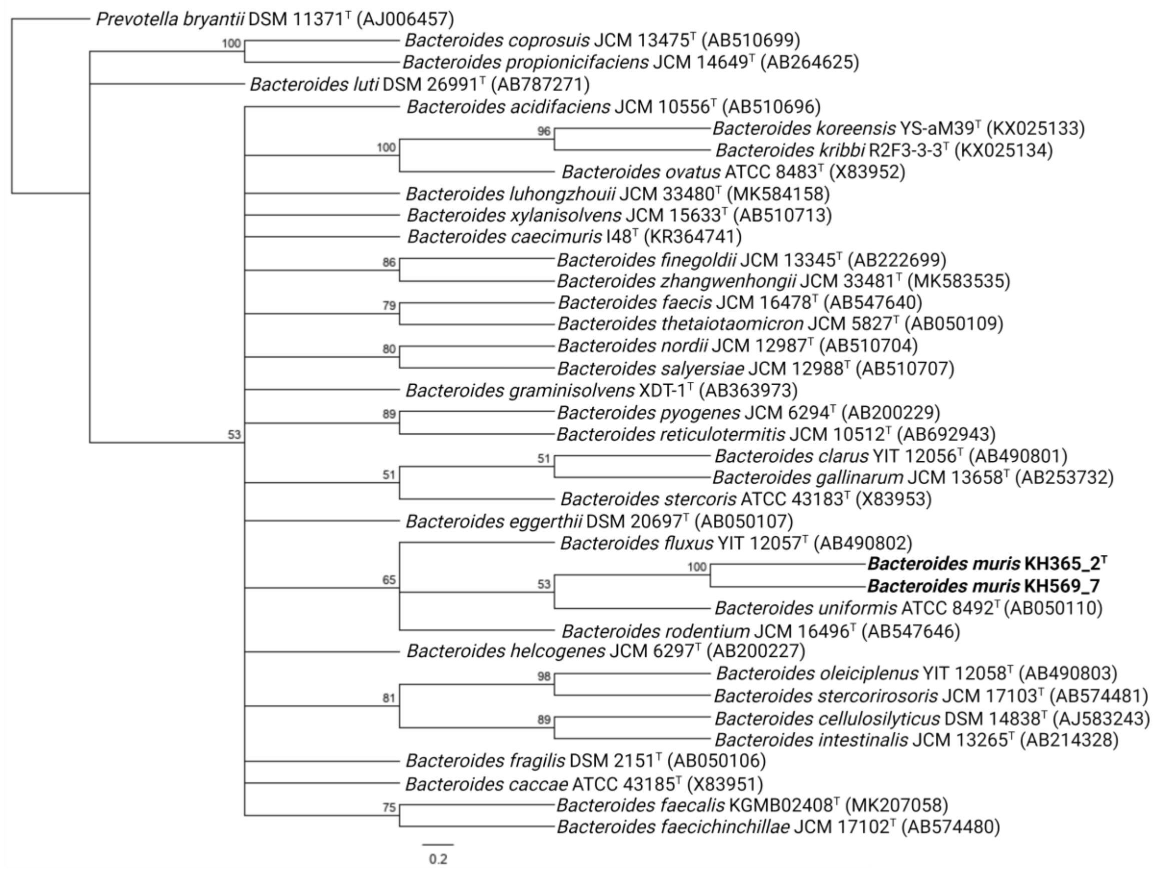

Fig. S1 Maximum-likelihood tree based on 16S rRNA gene sequences, showing the relatedness between *B. muris* strains KH569_7 and KH365_2^{T} (both in bold) and other members of the genus *Bacteroides*. The accession numbers of the 16S rRNA gene sequences are indicated in brackets. Numbers at nodes indicate bootstrap values (>50%) calculated from 1000 trees. *Prevoltella bryantii* DSM 11371^{T} was used as outgroup to root the tree. The bar represents substitutions per nucleotide position.

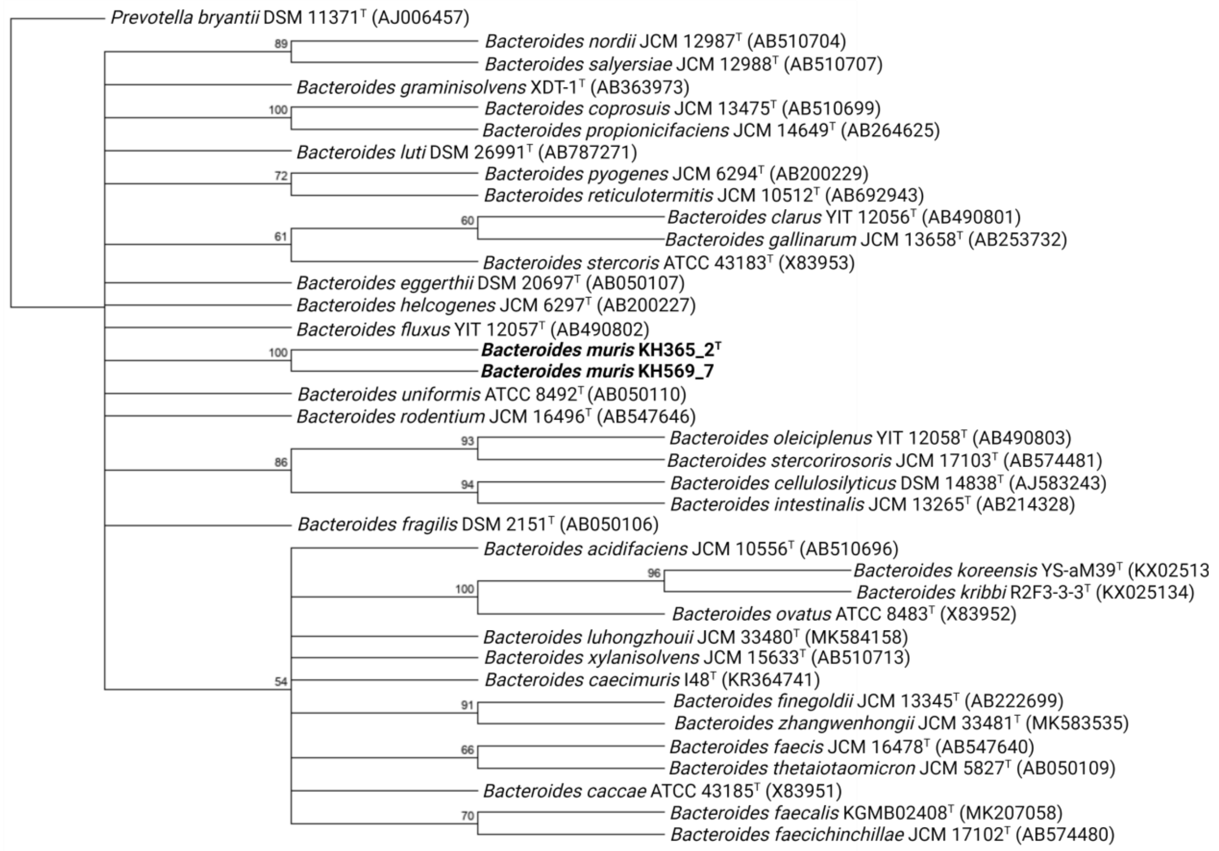

Fig. S2 Maximum-parsimony tree based on 16S rRNA gene sequences, showing the relatedness between *B. muris* strains KH569_7 and KH365_2^T (both in bold) and other members of the genus *Bacteroides*. The accession numbers of the 16S rRNA gene sequences are indicated in brackets. Numbers at nodes indicate bootstrap values (>50%) calculated from 1000 trees. *Prevoltella bryantii* DSM 11371^T was used as outgroup to root the tree.

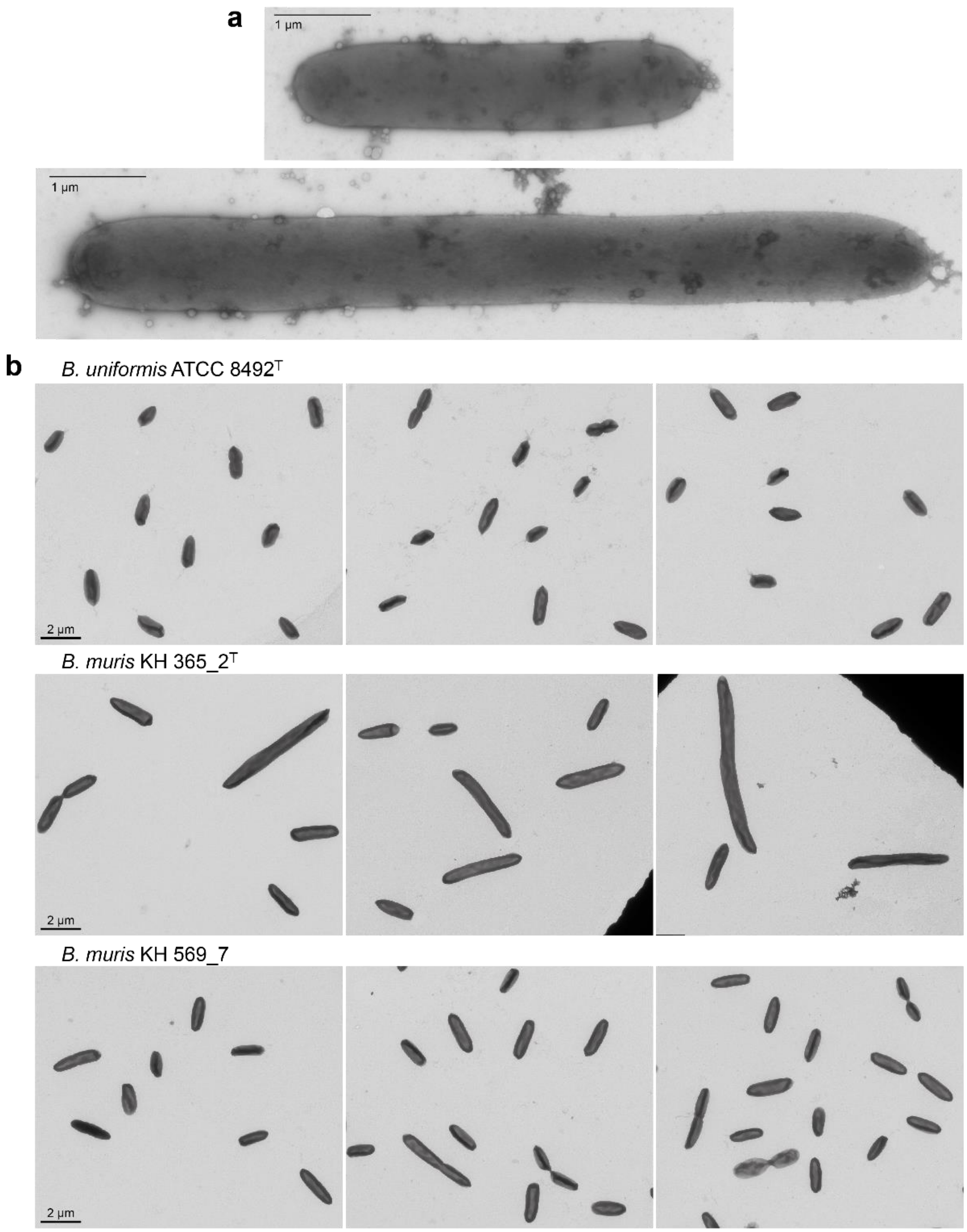

Fig. S3 Transmission electron micrographs of whole bacteria. (a) Negatively stained bacteria from a fresh culture of *B. muris* KH365_2^T. In this strain, unusually long bacteria were occasionally observed, in contrast to the other two strains. Samples were prepared as in Fig. 2c. (b) Comparison of the variation in bacterial size between the three strains of bacteria. Bacterial cultures were fixed overnight with 1% glutaraldehyde in 200 mM HEPES, pH 7.4. Fixed bacteria were pelleted and resuspended at a higher density than the original culture in order to increase the density of bacteria on grids. Grids were stained with 1% uranyl acetate for 2 min, washed with dH_2O and air-dried. In this way, unbound electron dense stain was prevented from accumulating around bacteria and enlarging their profiles.

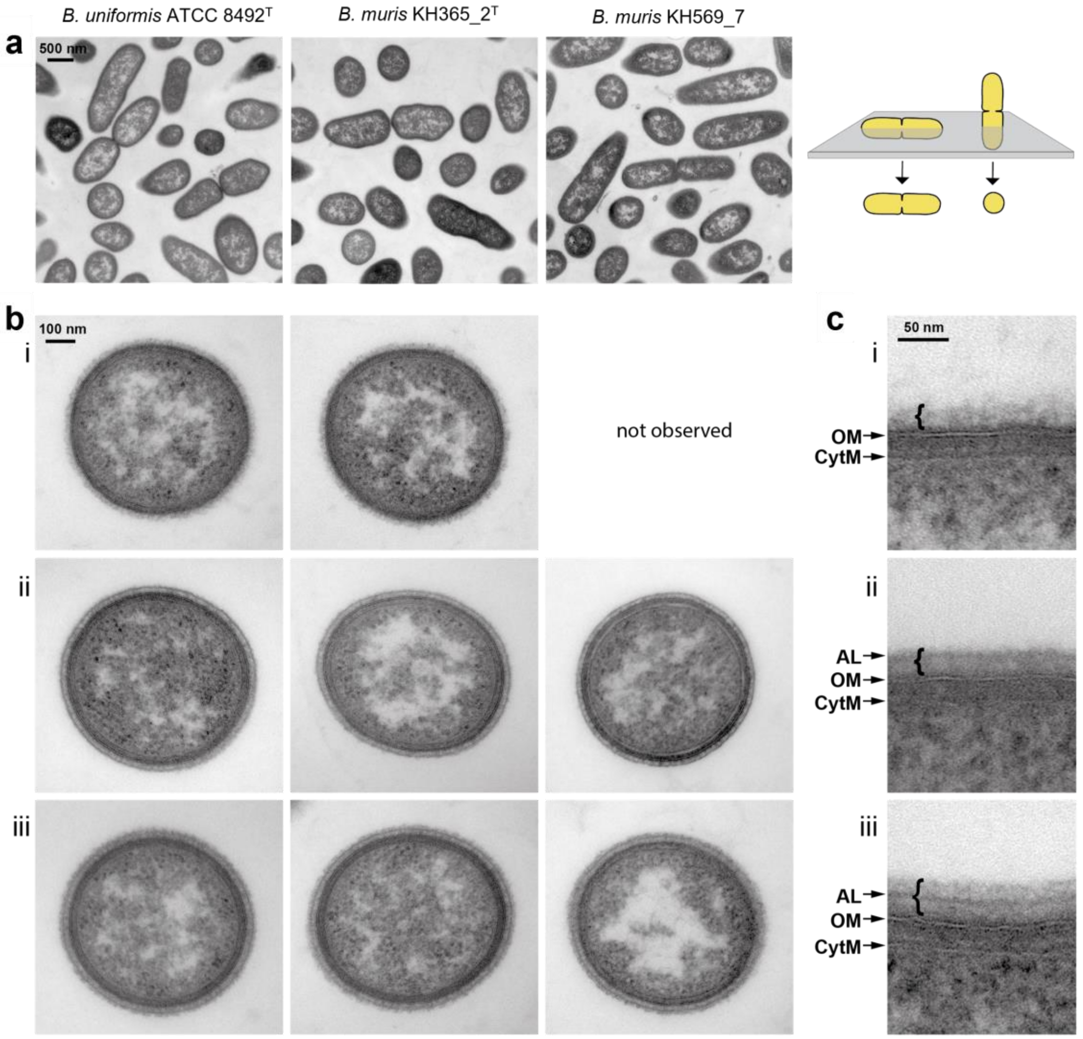

Fig. S4 Ultrastructural analysis of *B. uniformis* ATCC 8492[T], and the newly described strains *B. muris* KH 365_2[T] and KH 569_7. Thin sections of resin embedded bacteria were imaged by transmission electron microscopy. (a) Shape of cell profiles depends on the orientation of the sectioning plane through a bacterium. Bacteria sectioned in longitudinal and in transverse orientation appear elongated and circular respectively (scheme). (b) Comparison of the structure of the cell envelope seen on transverse sections. In addition to the two bilayered membranes characteristic for Gram-negative bacteria, the capsule-like structure lies external to the outer membrane. Variation in the appearance of the capsule was observed in all three strains. (c) Detailed view of the cell envelope illustrating variation in the microcapsule (bracket): (i) one fringed layer, (ii) an electron dense layer with an additional, peripheral compact layer (AL), or (iii) containing three layers, including a fringed layer beyond the AL (bottom). CytM, cytoplasmic membrane; OM, outer membrane. All three images were taken on longitudinal sections of *B. muris* KH 365_2[T].

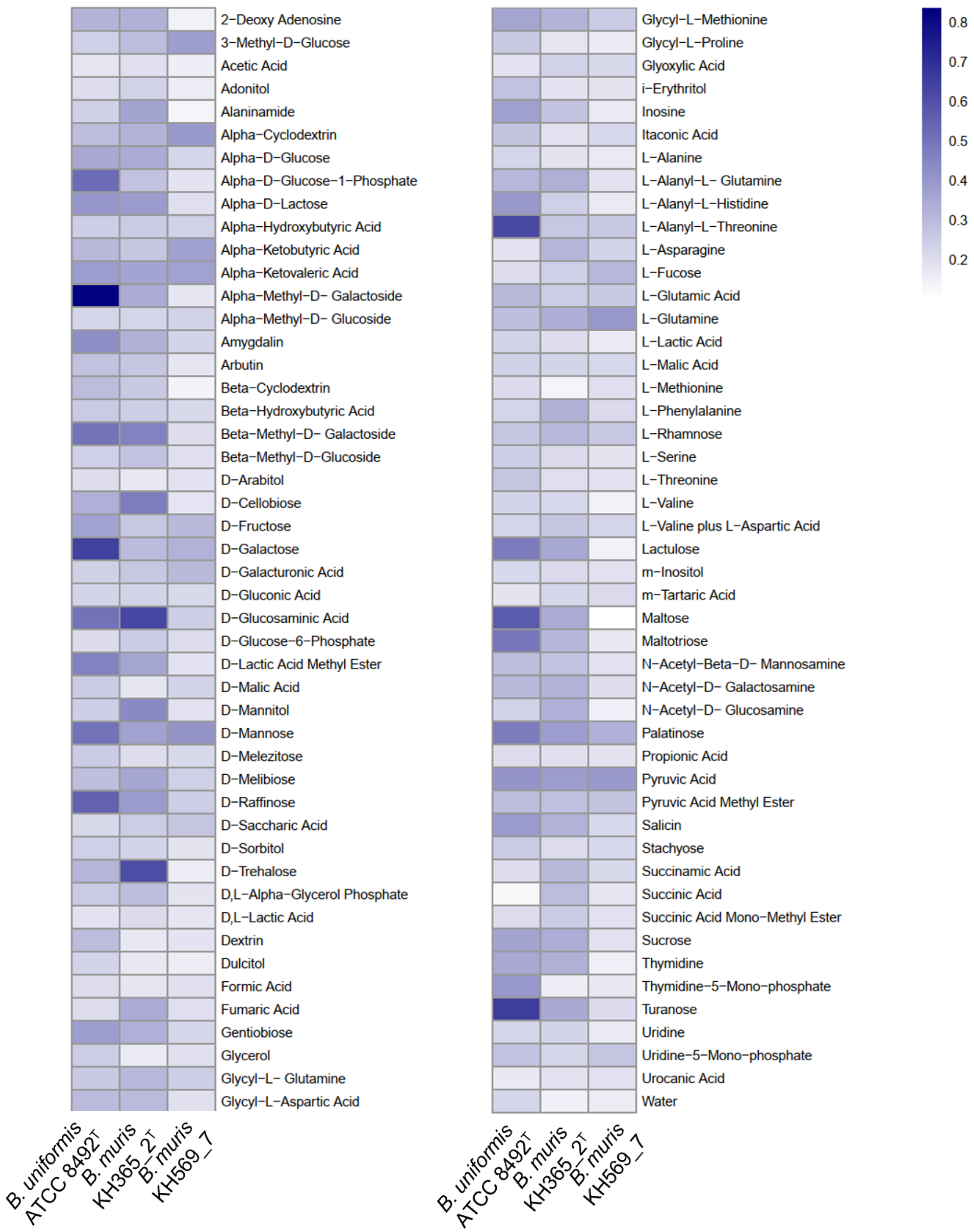

Fig. S5 Heatmap showing the utilization patterns of different metabolites by *B. muris* KH 365_2^T, *B. muris* KH 569_7 and *B. uniformis* ATCC 8492^T strains. The shade of color on both heatmaps represents the difference between the final and initial turbidity, ΔT (590 nm) from the Biolog assay. The mean from of three independent experiments is shown.

Table S1. Average nucleotide identity (orthoANIu) and digital DNA-DNA hybridization (dDDH) values of *B. muris* KH365_2^T, *B. muris* KH569_7 and closely related *Bacteroides* strains.

**Strains**	**Genbank accession No.**	**orthoANIu (%)**		**dDDH (%) ***		**G+C (% mol)**
		KH365_2^T	KH569_7	KH365_2^T	KH569_7	
KH365_2^T	JAMZED000000000	–	**98.65**	–	**87.0**	46.02
KH569_7	JAMZEE000000000	**98.65**	–	**87.0**	–	46.03
B. uniformis ATCC 8492^T	GCA_000154205	92.01	91.85	49.2	48.6	46.45
B. rodentium JCM 16496^T	GCA_000614125	90.44	90.47	43.2	43.7	47.05
B. fluxus YIT 12057^T	GCA_000195635	80.07	80.22	24.6	24.4	45.57

* Results are percentages based on calculations using Formula 2: The sum of all identities found in high-scoring segment pairs (HSPs) were divided by the overall HSP length. Formula 2 is independent of genome length and thus is more robust when applied to incomplete draft genomes (Meier-Kolthoff et al. 2013b).

Table S2. Average nucleotide identity (orthoANIu), digital DNA-DNA hybridization (dDDH) of *B. muris* KH365_2^T, *B. muris* KH569_7 and *Bacteroides* sp. NM69_E16B and their identity to other closely related species.

**Strain**	**Genbank accession No.**	**orthoANIu (%)**	**dDDH (%) ***	**Related genomes (genome ID, species name, ANI [%]) ****
		NM69_E16B		
KH365_2^T	JAMZED000000000	98.03	83.7	GCF_000154205.1, *Bacteroides uniformis*, 92.44; GCF_000614125.1, *Bacteroides rodentium*, 90.86; GCF_000195635.1, *Bacteroides fluxus*, 81.67
KH569_7	JAMZEE000000000	98.06	83.4	GCF_000154205.1, *Bacteroides uniformis*, 92.29; GCF_000614125.1, *Bacteroides rodentium*, 90.93; GCF_000195635.1, *Bacteroides fluxus*, 81.58
NM69_E16B	GCA_004793475.1	–	–	GCF_000154205.1, *Bacteroides uniformis*, 92.15; GCF_000614125.1, *Bacteroides rodentium*, 90.91; GCF_000195635.1, *Bacteroides fluxus*, 81.55

* Results are percentages based on calculations using Formula 2: The sum of all identities found in high-scoring segment pairs (HSPs) were divided by the overall HSP length. Formula 2 is independent of genome length and thus is more robust when applied to incomplete draft genomes (Meier-Kolthoff et al. 2013).

** Results were obtained using the Genome Taxonomy Database Toolkit(Chaumeil et al. 2019).

Table S3. Comparison of the genomic features of strains KH365_2^T, KH569_7, and closely related species in the genus *Bacteroides*

Strains: 1, *B. muris* KH365_2^T; 2, *B. muris* KH569_7; 3, *B. uniformis* ATCC 8492^T; 4, *B. rodentium* JCM 16496^T; 5, *B. fluxus* YIT 12057^T

	**1**	**2**	**3**	**4**	**5**
Genbank accession No.	JAMZED000000000	JAMZEE000000000	GCA_000154205	GCA_000614125	GCA_000195635
Number of contigs	232	165	49	148	117
Size (Mb)	4.2	4.2	4.8	4.9	4.3
CDS	3886	3903	4663	4926	3921
rRNAs	6	5	13	3	3
tRNAs	66	48	63	57	58
Other RNAs	2	1	2	2	2

References

Chaumeil P-A, Mussig AJ, Hugenholtz P, Parks DH (2019) GTDB-Tk: a toolkit to classify genomes with the Genome Taxonomy Database. Bioinformatics. https://doi.org/10.1093/bioinformatics/btz848

Meier-Kolthoff JP, Auch AF, Klenk H-P, Göker M (2013a) Genome sequence-based species delimitation with confidence intervals and improved distance functions. BMC Bioinformatics 14:60. https://doi.org/10.1186/1471-2105-14-60
